# Real-Time Target Detection Method Based on Lightweight Convolutional Neural Network

**DOI:** 10.3389/fbioe.2022.861286

**Published:** 2022-08-16

**Authors:** Juntong Yun, Du Jiang, Ying Liu, Ying Sun, Bo Tao, Jianyi Kong, Jinrong Tian, Xiliang Tong, Manman Xu, Zifan Fang

**Affiliations:** ^1^ Key Laboratory of Metallurgical Equipment and Control Technology of Ministry of Education, Wuhan University of Science and Technology, Wuhan, China; ^2^ Research Center for Biomimetic Robot and Intelligent Measurement and Control, Wuhan University of Science and Technology, Wuhan, China; ^3^ Hubei Key Laboratory of Mechanical Transmission and Manufacturing Engineering, Wuhan University of Science and Technology, Wuhan, China; ^4^ Precision Manufacturing Research Institute, Wuhan University of Science and Technology, Wuhan, China; ^5^ Hubei Key Laboratory of Hydroelectric Machinery Design & Maintenance, China Three Gorges University, Yichang, China

**Keywords:** Deep learning, target detection, MobileNets-SSD, depthwise separable convolution, residual module

## Abstract

The continuous development of deep learning improves target detection technology day by day. The current research focuses on improving the accuracy of target detection technology, resulting in the target detection model being too large. The number of parameters and detection speed of the target detection model are very important for the practical application of target detection technology in embedded systems. This article proposed a real-time target detection method based on a lightweight convolutional neural network to reduce the number of model parameters and improve the detection speed. In this article, the depthwise separable residual module is constructed by combining depthwise separable convolution and non–bottleneck-free residual module, and the depthwise separable residual module and depthwise separable convolution structure are used to replace the VGG backbone network in the SSD network for feature extraction of the target detection model to reduce parameter quantity and improve detection speed. At the same time, the convolution kernels of 1 × 3 and 3 × 1 are used to replace the standard convolution of 3 × 3 by adding the convolution kernels of 1 × 3 and 3 × 1, respectively, to obtain multiple detection feature graphs corresponding to SSD, and the real-time target detection model based on a lightweight convolutional neural network is established by integrating the information of multiple detection feature graphs. This article used the self-built target detection dataset in complex scenes for comparative experiments; the experimental results verify the effectiveness and superiority of the proposed method. The model is tested on video to verify the real-time performance of the model, and the model is deployed on the Android platform to verify the scalability of the model.

## 1 Introduction

With the appearance and progress of powerful hardware devices such as image processors, deep learning has achieved rapid development. In recent years, deep convolutional neural networks have been widely applied to solve various tasks of computer vision. Traditional visual tasks include image classification, location, detection, and segmentation (Evan, et al., 2017; Jiang et al., 2019a; He, et al., 2019). In traditional visual tasks, feature extraction, a complicated task, has been completely replaced by convolutional neural networks (Sun, et al., 2020; Tian, et al., 2020; Liu, et al., 2021a; Liao, et al., 2021). On this basis, deep learning technology can improve the visual tasks of most complex scenes (Li, et al., 2019a; Jiang, et al., 2019b; Huang, et al., 2022). For example, automatic driving, face monitoring, pedestrian tracking, and so on are all tasks in very complex scenes, but the current research mostly focuses on how to improve the accuracy of target detection technology, which leads to the excessively large target detection model to a certain extent (Chen, et al., 2021a; Bai, et al., 2021; Duan, et al., 2021).

Target detection methods based on deep learning developed rapidly after 2012, which can be roughly divided into two categories: one is a two-stage model, which divides target detection into two stages: candidate box selection and target classification; the other is a one-stage model, which treats classification and localization as regression tasks. The two-stage target detection model first determines whether the target exists in the candidate region, and then determines the category with the classifier. However, most of the current research focuses on how to improve the accuracy of target detection technology, which leads to the excessively large target detection model to a certain extent. It is still challenging to synchronously realize high detection accuracy and real-time performance of objects in complex scenes.

This article proposes a real-time target detection method based on a lightweight convolutional neural network to reduce the parameters of the target detection model and improve the detection speed. First, Kinect is used to establish the target detection dataset in complex scenes, and the existing lightweight network is comprehensively studied. Then, combined with the depthwise convolution and bottleneck-free residual module, the depthwise residual module is proposed, and the MobileNet-SSD network is further improved by using the deep separable residual module, deep separable convolution, and convolution substitution structure. A real-time target detection model based on a lightweight convolutional neural network is established. The effectiveness of the proposed method is verified by comparing the established dataset with the existing lightweight target detection algorithm. Finally, the real-time detection model is tested on video, and the model is deployed to the mobile terminal to verify the scalability of the model.

The key contributions of this work are:

1) Combining depth-separable convolution and bottle-free residual module, the depth-separable residual module is proposed.
2) The MobileNet-SSD network is further improved by using the depthwise separable residual module, depthwise separable convolution, and convolutional substitution structure, and a real-time target detection method based on a lightweight convolutional neural network is proposed.
3) Target detection datasets are established in complex scenarios
4) Multiple groups of comparative experiments are conducted, and the proposed method is used to detect the video to verify the real-time performance of the model.

The rest of this article is organized as follows: Section 2 discusses the related work of target detection, followed by a target detection method based on improved MobileNet-SSD in Section 3. A comparative experiment is carried out using self-built datasets in Section 4. Section 5 concludes the paper with a summary and future research directions.

## 2 Related Work

At present, the mobile intelligent terminal has gradually become a necessity in people’s life (Li, et al., 2019b; Hu, et al., 2019; Yu, et al., 2019; Cheng et al., 2021; Jiang, et al., 2021c; Huang, et al., 2021); while the mobile intelligent device for embedded devices is limited by the storage and computing power, the development of technology, such as unmanned drones, also need terminal real-time feedback image- and video-processing results; thus, the target detection model size and the complexity of calculation are difficult requirements (Luo, et al., 2020; Liu, et al., 2021b; Sun, et al., 2021; Liu, et al., 2022).

The task of target detection is to classify objects in the image and further determine their position in the image. For the recognition task, the network needs to extract deeper semantic features, that is, the essence of the target features, so as to distinguish between the target objects and improve the accuracy of recognition. For positioning tasks, location information needs to be saved as much as possible to bring the detection frame closer to the actual position of the target object in the image.

The traditional target detection process is as follows: first, multiple image regions with possible target objects are selected by sliding windows of different sizes; then, feature extraction methods such as SIFT (scale-invariant feature transform) and HOG (histogram of oriented gradient) are used to transform the information contained in the region into feature vectors and then classify them, commonly using the support vector machine (SVM) classifier. The DPM (deformable parts model) was proposed in 2010, which decomposes the target object into various parts for training and merges the prediction results of all parts during prediction to complete the detection of the target object. However, since the traditional target algorithm extracts the candidate region information and manually designs the features, the application range has great limitations. For example, the Haar feature is suitable for face detection, and the detector trained by this feature cannot detect other types of targets. In addition, the traditional target detection algorithm generates multiple candidate regions through traversal, which takes a lot of time. In addition, the traditional target detection algorithm classification training detector may produce the problem of feature vector “dimension disaster.”

Ross et al. proposed an R-CNN object detection model based on convolutional neural networks (CNNs), which first used depth to detect objects. However, the scaling of candidate regions has certain limitations in detection accuracy, and the training of this algorithm is complicated. In 2015, He et al. proposed the SPP-NET model to transform feature information of candidate regions of arbitrary size into feature vectors of fixed length. In the same year, Girshick proposed the fast R-CNN algorithm, which was based on ROI pooling (region of interest pooling), fixed the feature length of candidate regions, and used the multi-task loss function for training, which improved the training and detection efficiency of the target detection algorithm. In order to achieve real-time detection, researchers use the integrated convolutional neural network to complete target detection and improve the detection efficiency of the algorithm. Regression-based algorithms of YOLO and SSD (single-shot multibox detector) have continually appeared. However, both SSD and YOLO only use the characteristic information of a single scale for prediction, and the detection accuracy of multi-scale targets and small objects is low.

Due to the diversity of application scenarios of target detection technology (Sun, et al., 2022a; Weng, et al., 2021; Yun, et al., 2022; Zhao, et al., 2021), target detection algorithm should realize the lightweight of the model, solve the efficiency problem of the model, and successfully deploy or apply to mobile devices, industrial computers and other embedded platforms (Xiao, et al., 2021; Yang, et al., 2021; Sun, et al., 2022b; Liu et al., 2021c). Therefore, the lightweight target detection model has become another hot issue (Ma, et al., 2020; Liu, et al., 2021d). He et al. (2015) used the lightweight deep separable residual network as the basic network of fast R-CNN to reduce the parameters of the network model, fused the multi-layer convolution features in the basic network after local response normalization, enhanced the completeness of target feature information, and trained the network model in combination with Softmax loss function and central loss function so that the network model could learn other different target characteristics. Ren and Bao (2020) reduced the amount of network computation by using MobileNet as the basic network and replacing the standard convolution in the SSD detection layer with the inverse residual convolution. Evan et al. (2017) reduced darknet53, the backbone network of YOLOv3, and added an improved dense connection network and spatial pyramid pooling on the backbone network, which greatly improved the speed at the expense of accuracy. Zhao et al. (2020) integrated a 5 × 5 depthwise separable convolution kernel on the basis of the MobileNetV2-SSD Lite model to further improve the recognition accuracy of the algorithm for small target objects, and the experimental results show that LMS-DN only needs fewer parameters and calculation costs to obtain higher identification accuracy and stronger anti-interference than other popular object detection models. Zhang et al. (2021) proposed a lightweight target detection network MN-YOLO (MobileNet-YOLOv4-tiny) suitable for embedded platforms using depthwise separable convolution instead of standard convolution to reduce the number of model parameters and calculations; at the same time, the visible light target detection model is used as the pretraining model of the infrared target detection model and the infrared target dataset collected on the spot is fine-tuned to obtain the infrared target detection model. Currently, miniaturized versions of YOLO and SSD algorithms are commonly used on embedded platforms (Alex, et al., 2017; Cao, et al., 2018; Cheng, et al., 2020; Chen, et al., 2021c; Hao, et al., 2021). The research of the MobileNet-SSD network framework to realize network model compression and multi-scale target detection is increasing gradually. Based on the Mobilenet-SSD framework, Li et al. (2019c) used the time characteristics of video to effectively improve the confidence level of detection and enhance the stability of detection, which provides a certain reference value for unmanned target detection. Although these algorithms have low computational load and fast detection speed, their detection accuracy is generally low, making it difficult to achieve a balance between computational load and accuracy (Jiang, et al., 2019d; Jiang et al., 2019e; Huang, et al., 2019; Li, et al., 2020).

To sum up, there are many algorithms for target detection at present, but the problems of target detection accuracy, model size, and detection speed still need to be solved in the application scenarios of service robots and other mobile devices (Sandler, et al., 2018; Qiu, et al., 2019; Meng, et al., 2020; Yu et al., 2020; Li, et al., 2021; Tao et al., 2022a). Therefore, a real-time target detection method based on a lightweight convolutional neural network is proposed in this article to reduce the number of target detection model parameters and improve the detection speed.

## 3 Improved MobileNet-SSD Network

### 3.1 SSD

**FIGURE 1:**
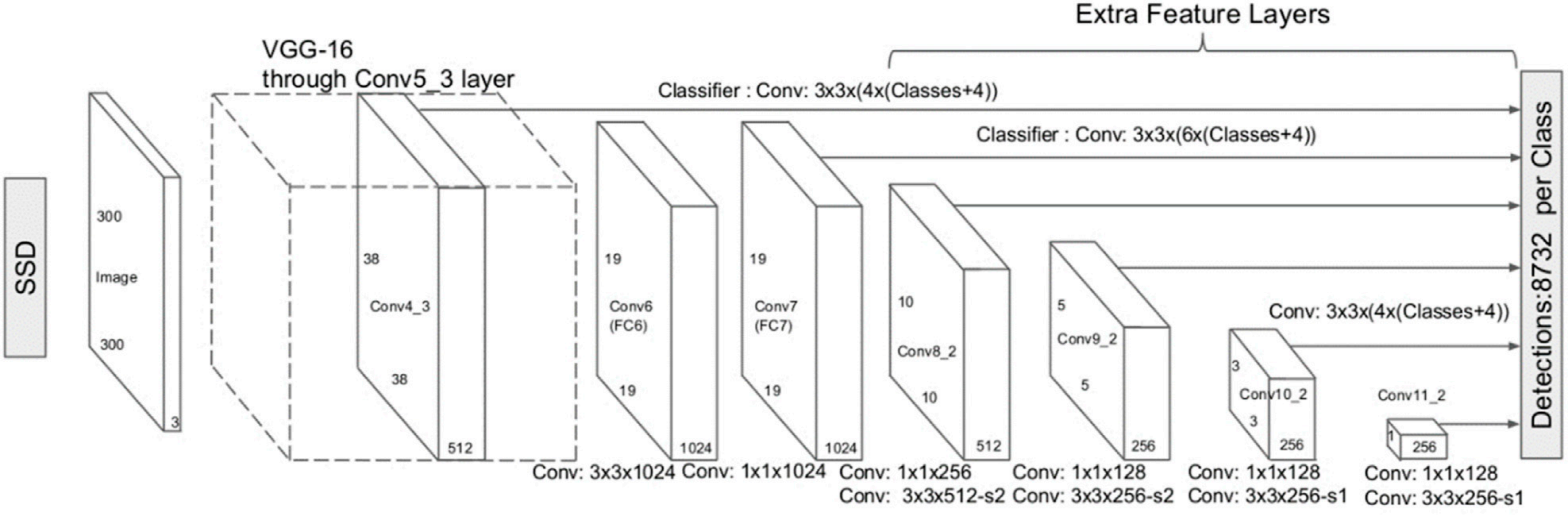
SSD network structure.

SSD is a one-stage target detection algorithm (Tan, et al., 2020; Wu, et al., 2022), which directly generates the category probability and position coordinate value of objects. After a single detection, the final detection result can be directly obtained, so it has a faster detection speed. The network detection framework is shown in Figure 1. Traditional SSD uses VGG16 as the feature extraction network. The full connection layer of VGG16 is removed and the convolution layer is added to obtain more multi-layer feature maps for detection. At the same time, SSD makes full use of multi-level feature maps in the classification regression network, and the corresponding classification layer of all level feature maps shares weights with the location regression layer.

One of the cores of SSD is to detect objects of different sizes using feature maps of different levels, that is, to extract targets using feature maps output by each convolution layer. The scale of the anchor frame corresponding to the bottom-level feature graph to the high-rise feature graph is linearly divided from small to large. Steps for generating anchor frame are as follows:

1) A set of concentric anchor frames is generated centering on the midpoint of each point on the feature graph.
2) m feature maps of different levels are used to extract targets. The scales of the bottom feature map corresponding to the anchor frame are $s_{\min}$ , and the scales of the top are $s_{\max}$ . That of the other layers are:

$$s_{k}=s_{\min}+\frac{s_{\min}-s_{\max}}{m-1}\left(k-1\right),k\in \left[1,m\right]$$ (1)

3) Different ratios [1, 2, 3, 1/2, and 1/3] were used to calculate the width and height of the anchor frame using Eqs 2, 3:

$$w_{k}^{a}=s_{k}\sqrt{a_{r}}$$ (2)

$$h_{k}^{a}=s_{k}/\sqrt{a_{r}}$$ (3)

4) In the case of ratio = 0, the specified scale is as follows:

$$s_{k}^{\prime }=\sqrt{s_{k}s_{k+1}}$$ (4)

### 3.2 MobileNet-SSD

**FIGURE 2:**
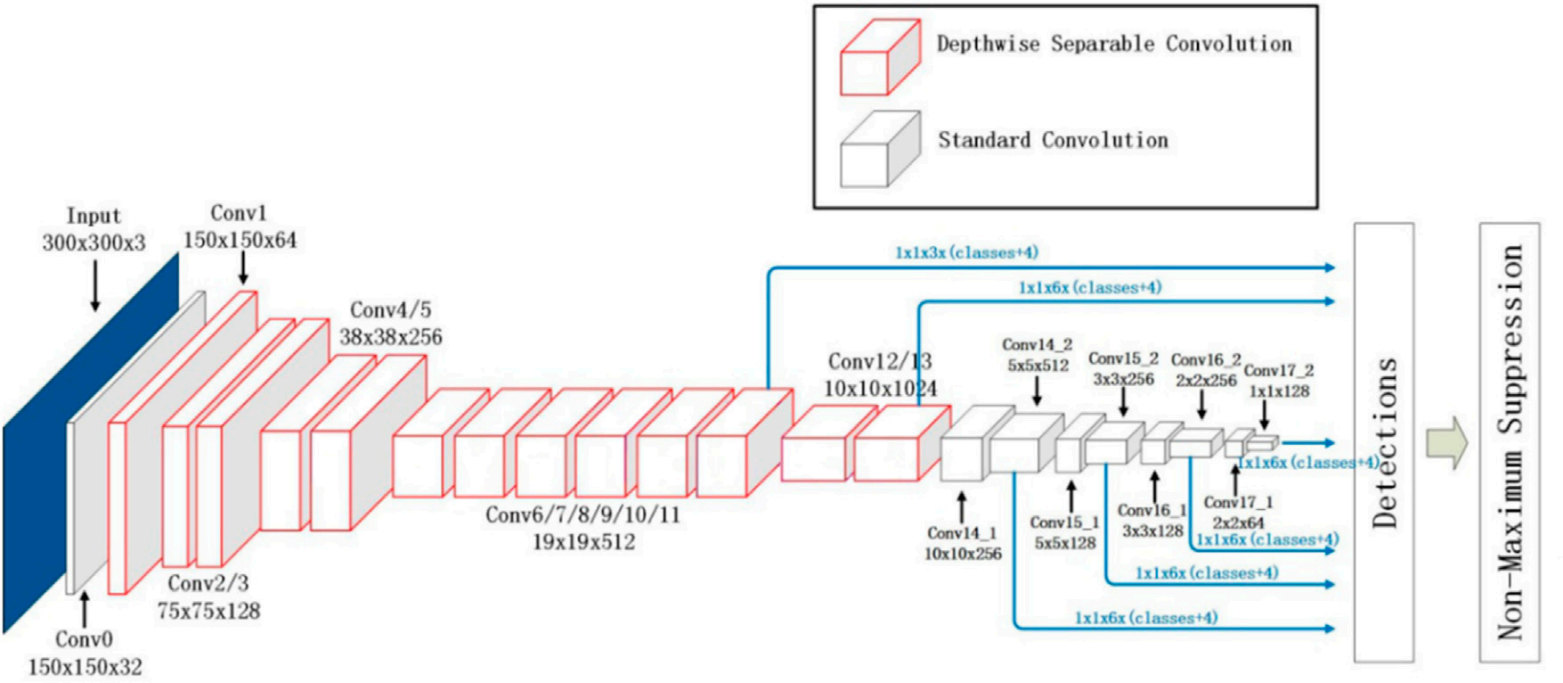
MobileNet-SSD network structure.

The network detection framework of MobileNet-SSD is shown in Figure 2 (Algarni, 2021). The front-end network of MobileNet VGG16 is replaced by MobileNet, and the global average pooling layer, full connection layer, and Sofamax layer of MobileNet network are removed, followed by the back-end detection network of SSD. A MobileNet-SSD network was formed. Because the front-end network of the MobileNet-SSD network was deeper than that of SSD, the depth of the whole model was larger than that of the SSD network. From the perspective of the SSD back-end detection network, both MobileNet-SSD and SSD networks were detected by extracting features from the feature map of six scales. Because the MobileNet-SSD network adopted depthwise separable convolution, the resolution of the feature map of the back-end detection network was only half of that of the SSD network. Therefore, the network had less computation and computational complexity.

### 3.3 Improved MobileNet-SSD

The core of MobileNet is to consider image regions and channels separately and use depthwise convolution to replace standard convolution. The process of standard convolution is divided into depthwise convolution and pointwise convolution, that is, each channel is first convolved, then the information between channels is fused by 1 × 1 convolution, the number of channels in the feature graph is changed, and the same effect as standard convolution is achieved (Liao, et al., 2020; Liu, et al., 2021e).

Depthwise separable convolution decomposes a complete convolution operation into two steps, that is, depthwise convolution and pointwise convolution. Different from conventional convolution operations, a convolution kernel of depthwise convolution is responsible for a channel, and a channel is convolved by only one convolution kernel. In the aforementioned conventional convolution, each convolution kernel operates on each channel of the input image simultaneously. Similarly, for a 128 × 128 pixel, three-channel color input image (128 × 128 × 3), depthwise convolution intially goes through the first convolution operation. Different from the aforementioned conventional convolution, depthwise convolution is completely carried out on a two-dimensional plane. The number of convolution kernels is the same as the number of channels in the upper layer, that is, channels and convolution kernels correspond one to one. The operation of pointwise convolution is similar to that of conventional convolution operation. The size of its convolution kernel is 1×1×M , and M is the number of channels in the upper layer. The convolution operation here will combine the feature graph of the previous step in the direction of the channel to generate a new feature graph.

**FIGURE 3:**
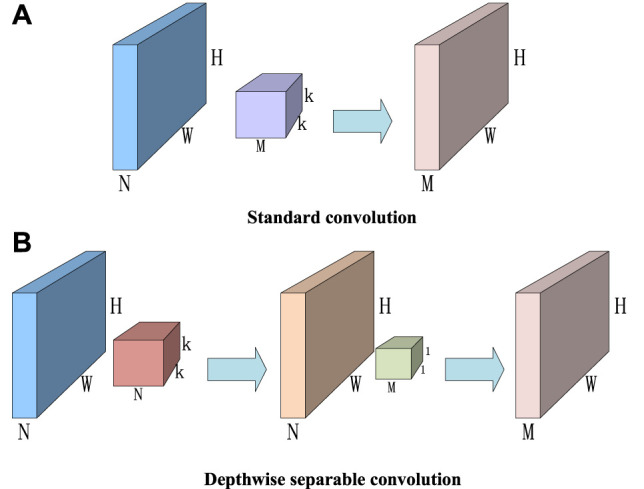
Standard convolution and depthwise separable convolution. **(A)** Standard convolution. **(B)** Depthwise separable convolution.

The structure of standard convolution and depth-separable convolution is shown in Figure 3 (Liu, et al., 2021; Li, et al., 2019; Hao, et al., 2021), where the input image dimension is H×W×N and the output image dimension is H×W×M . The standard convolution can be obtained through the convolution kernel of k×k×M , and the required number of parameters is N×k×k×M , while the depth-separable convolution is adopted. First, each channel of the input image is convolved, that is, the convolution kernel is k×k×N , and the required number of parameters is N×k×k . M convolution 1 × 1 is used to check the features of each channel for fusion. The number of parameters in this step is, N × k × k then the ratio of the number of parameters between the depthwise separable convolution and the standard convolution is shown in Eq. 5. When k=3 , the number of parameters of the depthwise separable convolution relative to the standard convolution is reduced by at least 8 to 9 times.

$$\frac{N\times k\times k+N\times 1\times 1\times M}{N\times k\times k\times M}=\frac{1}{M}+\frac{1}{k^{2}}$$ (5)

Two hyperparameters are set in MobileNet (Huang, et al., 2021), namely, the width multiplier and the resolution multiplier. The width multiplier controls the number of channels in the feature graph; when the width multiplier is less than 1, the model becomes thinner; the resolution multiplier is used to control the size of the feature graph, and both can reduce the number of parameters of the convolution flexibly. On the basis of MobileNet, MobileNetv2 uses an inverted residual block (Liu, et al., 2021; Sun, et al., 2020). First, 1 × 1 convolution is used to improve the dimension of features, and then 3 × 3 depth-separable convolution is used to extract features. Then, 1 × 1 convolution is used to reduce dimensions.

The depthwise separable convolution network in MobileNet can greatly reduce the number of parameters in the network model. Therefore, the standard convolution in the VGG16 structure in SSD is replaced by the depthwise separable convolutional neural network. However, compared with the standard convolution, the network layers of the depthwise separable convolution are deeper. As the number of network layers increases, network performance degrades, that is, the detection accuracy begins to decline after reaching saturation. Therefore, in order to effectively solve the problem of network performance degradation, this article improved the MobileNet-SSD feature extraction network by combining the residual connection mode of the ResNet model and depthwise separable convolution.

**FIGURE 4:**
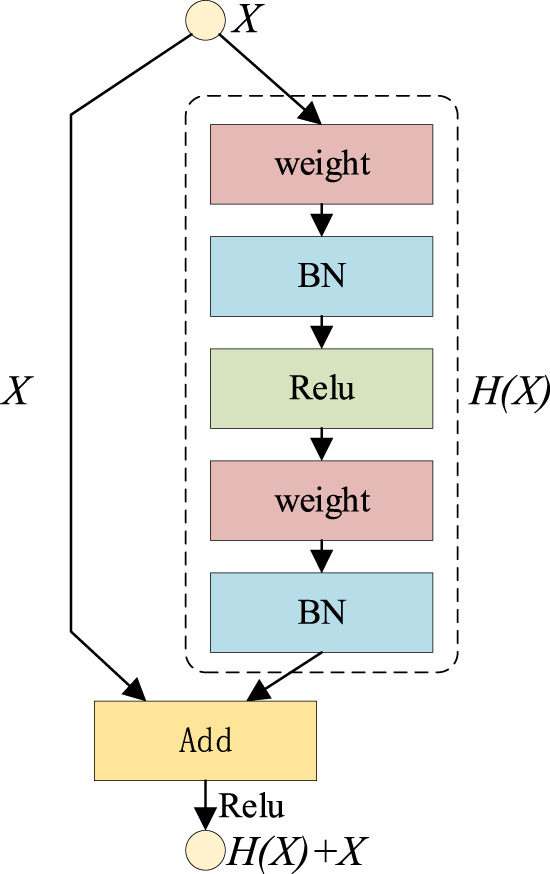
Residual learning.

If the input is set to X and a parametrized network layer is set to H , the output of this layer with X as the input will be H(X) . General CNN networks, such as VGG, can directly learn the expression of parameter function H through training, so as to directly learn X−>H(X) . Residual learning is committed to using multiple parametrized network layers to learn that the difference between input and output is (H(X)+X)−X . X is the direct mapping, while (H(X)+X)−X is the residual between input and output to be learned by the parameter network layer, and its principle is shown in Figure 4.

**FIGURE 5:**
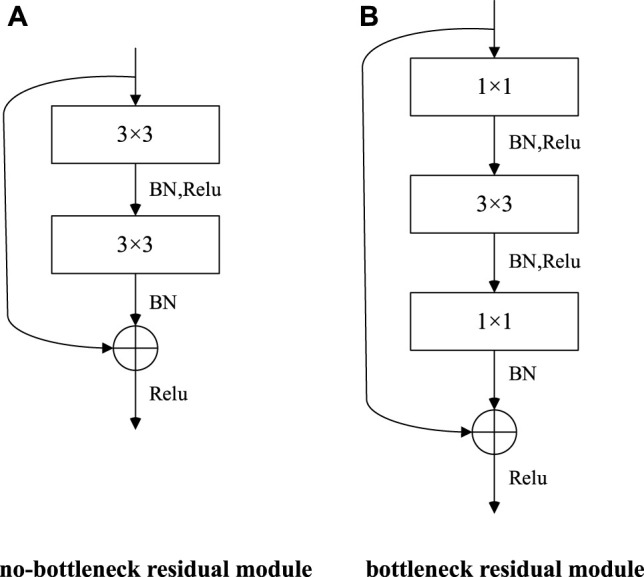
Two types of residual modules. **(A)** No-bottleneck residual module. **(B)** Bottleneck residual module.

The ResNet model has two types of residual modules, no-bottleneck residual module and bottleneck residual module, as shown in Figure 5.

BN and Relu shown in Figure 5 are the normalization layer and activation function, respectively, which help to speed up the training and generalization of the network model. Compared with the no-bottleneck residual module, the bottleneck residual module uses 1 × 1 convolution to reduce or expand the dimension of the feature graph, so that the 3 × 3 convolution is no longer affected by the number of channels’ input, and accordingly, the output of this module will not affect the next module. The model layers are deep, and the bottleneck-free module is beneficial to improve the model detection accuracy, while the bottleneck residual module is beneficial to improve the model running speed.

**FIGURE 6:**
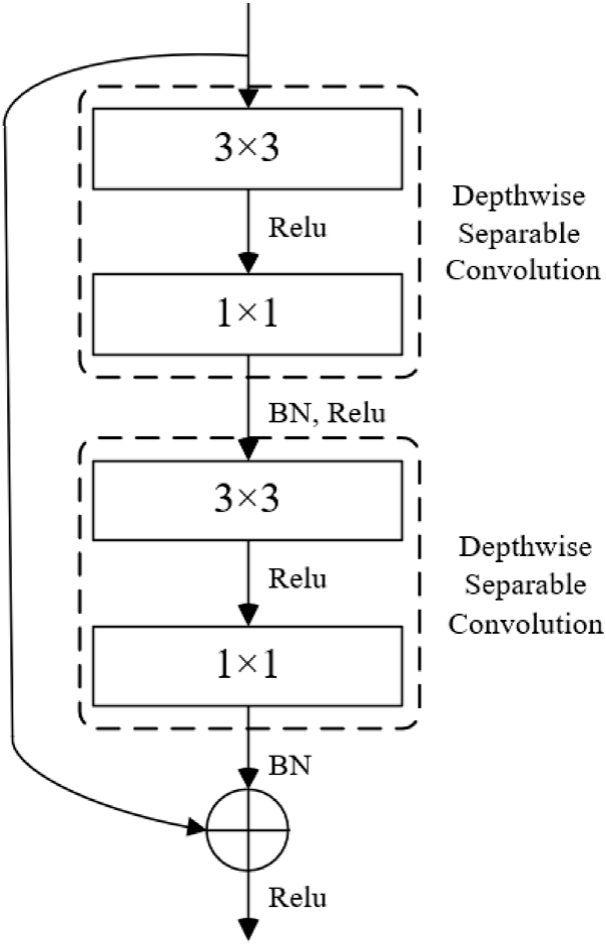
The depthwise separable residual module structure.

Compared with the combination of depthwise separable convolution and bottleneck residual module, the combination of depthwise separable convolution and bottleneck residual module has more obvious advantages in reducing the number of model parameters. Therefore, the depthwise separable convolution is combined with the bottleneck-free residual module to improve the feature extraction function of the trunk network. The structure of the combined depthwise separable residual module is shown in Figure 6. The network structure can effectively extract image feature information and greatly reduce the number of model parameters. Then, the module is combined with the depthwise separable structure to replace the VGG backbone network in the SSD network for feature extraction of the target detection model. Finally, for the network structure after Conv5_3 in SSD, the convolution sum of 1 × 3 and 3 × 1 convolution kernels are used to replace the standard convolution 3 × 3, thus obtaining multiple detection feature graphs corresponding to SSD.

Both the bottleneck residual module and the non-bottleneck residual module can reduce the number of parameters and computation by introducing depth-separable convolution. Table 1 compares the number of parameters of different types of residual modules when both input and output are 256 channels and 64 channels, respectively. In_Out_ C represents the number of input–output channels, Bt represents the bottleneck residual module, Non-Bt represents the non-bottleneck residual module, DS-Bt represents the separable bottleneck residual module after the introduction of depthwise separable convolution, and DS-Non-Bt represents the separable bottleneck residual module after the introduction of depthwise separable convolution. When the input and output are 64 channels, the number of Bt parameters is 4.35K, the parameter of DS-Bt is 2.77K, the parameter of Non-Bt is 36.86K, and the parameter of DS-Non-Bt is 4.67K. The parameter number of DS-Bt is 63.7% of that of Bt, and that of DS-Non-Bt is 12.7% of that of Non-Bt. When the input and output channels are 256 channels, the parameter number of Bt is 69.63K, that of DS-Bt is 35.65K, that of Non-Bt is 589.82K, and that of DS-Non-Bt is 67.84K. The number of parameters of DS-Bt is 51.2% of that of Bt, and that of DS-Non-Bt is 11.5% of that of Non-Bt. It can be seen from these data that the depth-separable convolution introduced by the bottleneck residual module has a higher benefit in reducing the number of parameters than the depth-separable convolution introduced by the bottleneck residual module. Moreover, the more channels there are, the more benefit can be obtained in reducing the number of parameters by introducing depthwise separable convolution.

**TABLE 1:** Number of module parameters with different residuals.

Residual block		Bt (K)	Non-Bt (K)	DS-Bt (K)	DS-non-Bt (K)
In_Out_C	64	4.35	36.86	2.77	4.67
	256	69.63	589.82	35.65	67.84

The specific parameters of lightweight SSD network structure based on depthwise separable convolution are shown in Tables 2 and 3, where Conv is the standard convolution, DW is the depthwise separable convolution, DS-RES is the depthwise separable residual module, and Alter Conv is the alternative convolution of corresponding parameters. The improved SSD adopts the idea of multi-layer feature detection in SSD. Multiple DS-RES modules are used to extract features, and use the feature graph of 19 × 19, 10 × 10, 5 × 5, 3 × 3, 2 × 2, and 1 × 1 for detection.

**TABLE 2:** The structure of a real-time target detection algorithm based on a lightweight convolutional neural network.

Network layer	Output size	Convolution Kernel size	Step
Input	300 × 300 × 3		
Conv1	150 × 150 × 32	3 × 3,32	2
DW1	150 × 150 × 64	3 × 3,64	1
DS-Res2	150 × 150 × 64	3 × 3,64 3 × 3,64	1
DW3	75 × 75 × 128	1 × 1,128	2
DW4	75 × 75 × 128	1 × 1,128	1
DS-Res5	75 × 75 × 128	3 × 3,128 3 × 3,128	1
DW6	38 × 38 × 256	1 × 1,256	2
DW7	38 × 38 × 256	1 × 1,256	1
DS-Res8	38 × 38 × 256	3 × 3,256 3 × 3,256	1
DW9	19 × 19 × 512	1 × 1,512	2
DW (10–14)	19 × 19 × 512	(1 × 1,256)×5	1
DS-Res15	19 × 19 × 512	3 × 3,512 3 × 3,512	1
DW16	10 × 10 × 1024	1 × 1,1024	2
DW17	10 × 10 × 1024	1 × 1,1024	1
Conv2	10 × 10 × 256	1 × 1,256	1
Alter Conv1	5 × 5 × 256	3 × 3,256	2
Conv3	5 × 5 × 128	1 × 1,128	1
Alter Conv2	3 × 3 × 256	3 × 3,256	2
Conv4	3 × 3 × 128	1 × 1,128	1
Alter Conv3	2 × 2 × 256	3 × 3,256	2
Conv5	2 × 2 × 64	1 × 1,64	1
Alter Conv5	1 × 1 × 128	3 × 3,128	2

**TABLE 3:** Parameters related to the experimental environment.

Category name	Parameter
operating system	Windows 10
CPU	AMD Ryzen 7
GPU	NVIDIA GeForce RTX 2070
Cuda with Cudnn	10.0/7.6.5
Python	3.6
Tensorflow, Keras	1.13.2/2.1.5
Opencv	4.5.1

The loss function is the weighted sum of position error and confidence error, as shown in Eq. 6.

$$L\left(x,c,l,g\right)=\frac{1}{N}\left(L_{conf}\left(x,c\right)+\alpha L_{loc}\left(x,l,g\right)\right)$$ (6)

where,

$$L_{loc}\left(x,l,g\right)=\sum_{i\in Pos}^{N}\sum_{m\in \left\{cx,cy,w,h\right\}}x_{ij}^{k}smooth_{L1}\left(l_{i}^{m}-g_{j}^{m}\right)$$ (7)

where,

$$g_{j}^{cx}=\frac{\left(g_{j}^{cx}-d_{i}^{cx}\right)}{d_{i}^{w}},\;\;\;g_{j}^{cy}=\frac{\left(g_{j}^{cy}-d_{i}^{cy}\right)}{d_{i}^{h}}$$ (8)

$$g_{j}^{w}=\log\left(\frac{g_{j}^{w}}{d_{i}^{w}}\right),\;\;\;g_{j}^{h}=\log\left(\frac{g_{j}^{h}}{d_{i}^{h}}\right)$$ (9)

where N is the number of prior frames of positive samples; α is the weight coefficient, set as 1; $x_{ij}^{p}\in \left\{0,\;1\right\}$ , when $x_{ij}^{p}=1$ , it means that the prior box i matches the target j and the target category is p ; c is the predicted value of category confidence; l is the position prediction value of prior frame; g is the location parameter of the real target; and $g_{j}^{cx}$ is the encoding of the real box.

The confidence error is a Softmax function:

$$L_{conf}\left(x,\;c\right)=-\sum_{i\in Pos}^{N}x_{ij}^{p}\log{\overset{\wedge }{c}}_{i}^{p}-\sum_{i\in Neg}\log{\overset{\wedge }{c}}_{i}^{o},\;\;where\;{\overset{\wedge }{c}}_{i}^{p}=\frac{\exp\left(c_{i}^{p}\right)}{\sum_{p}\exp\left(c_{i}^{p}\right)}.$$ (10)

## 4 Experiment and Analysis

### 4.1 Establishment of Target Detection Dataset in Complex Scenarios

**FIGURE 7:**
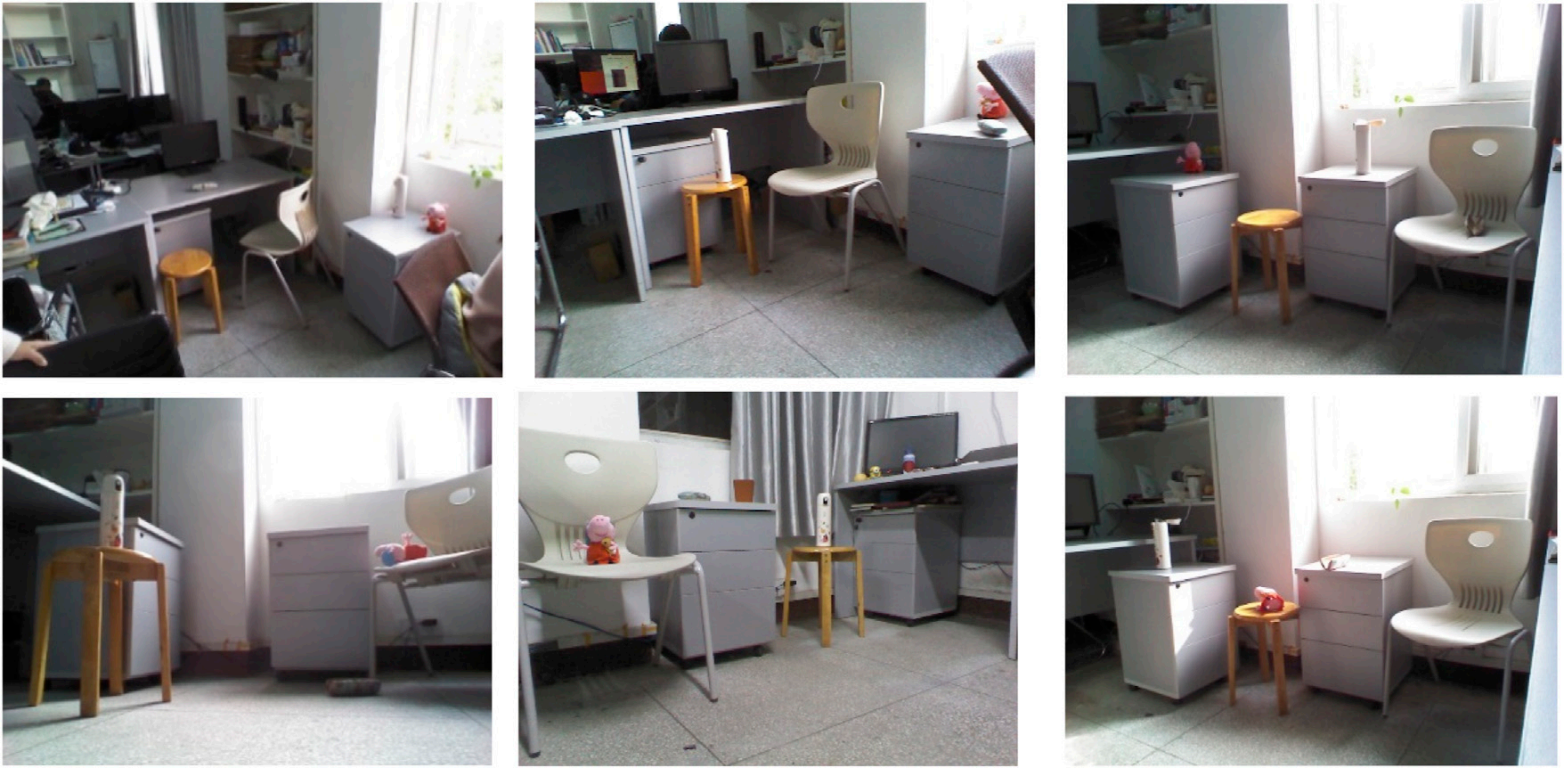
Color images of different angles, backgrounds, and lighting.

The Kinect camera was used to collect studio scenes in the manner of a video stream, and common objects in daily life were selected as detection targets, including toys, chair, stool, cabinet, glasses case, and cup. In the process of image collection, 1,064 color images of studio indoor scenes with different backgrounds, different light intensity, and different angles were collected, and the deformation of the toy page, thermos cup, and glasses case with different poses was taken into account. The chair and stool shape similarity improved the robustness of the target detection model. The collected pictures were named in one-to-one correspondence with four Arabic digits, and part of the sample of the indoor scene image constructed from this is shown in Figure 7.

Although the established image database contained images in various scenarios, the samples still lacked diversity. Therefore, on the basis of the established image data set, in order to increase the noise anti-interference ability of the model, the image of the dataset random chose some image processing operations; to make the data richer, each category contained a sample generally reaching equilibrium level so that it could be used to enhance the training dataset of the network, get better model performance, and improve the generalizability. Therefore, under the condition that other conditions remain unchanged, random rotation transform, inversion transform, image translation transform, noise disturbance, random clipping transform, image color transform, random occlusion, and random superposition of the aforementioned operations were carried out on the collected images to expand the dataset to 4,256 pieces. Label-Img was used to annotate the image dataset by category and position, and the indoor scene dataset was created.

### 4.2 Experiment and Result Analysis

**FIGURE 8:**
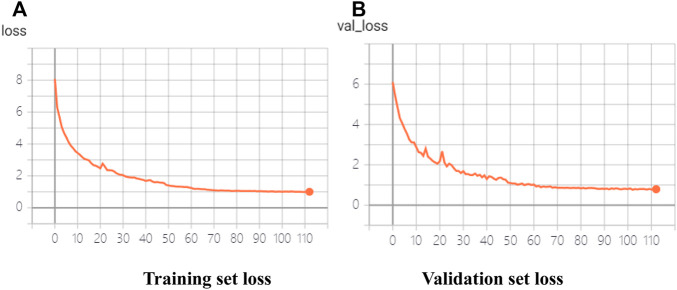
Training of trial target detection model based on a lightweight convolutional neural network. **(A)** Training set loss. **(B)** Validation set loss.

In this article, the improved MobileNet-SSD was trained by using the target detection dataset in complex scenarios. The parameter configuration of the experimental environment is shown in Tables 2 and 3. The Adam optimizer was used to adjust the learning rate during the training process. The training situations shown in Figures 8A,B represent the loss of the training set and verification set in the training process, respectively.

**FIGURE 9:**
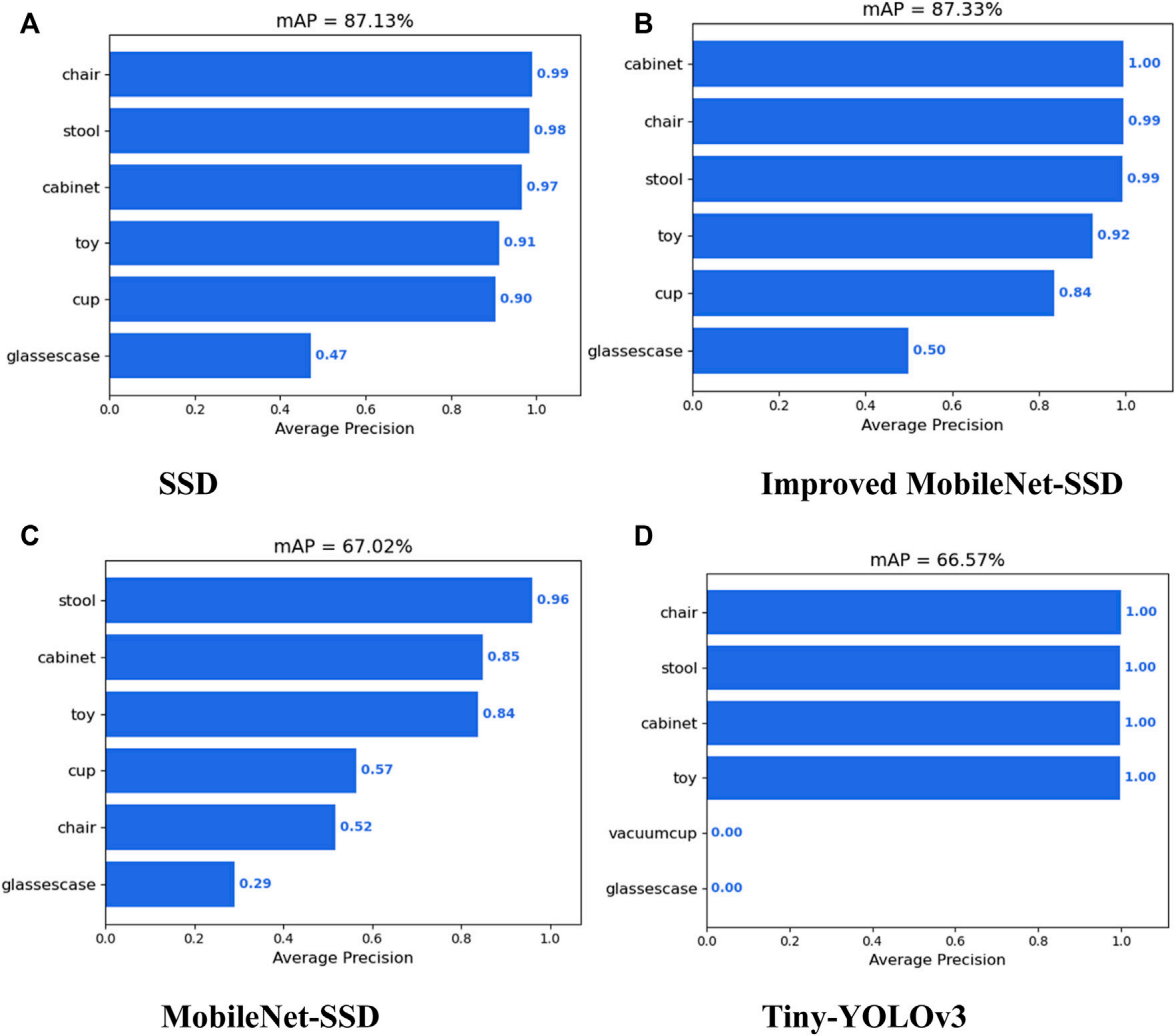
Comparison of detection accuracy between SSD and lightweight target detection algorithms for various classes. **(A)** SSD. **(B)** Improved MobileNet-SSD. **(C)** MobileNet-SSD. **(D)** Tiny-YOLOv3.

The comparative experiment is conducted on SSD, Tiny-Yolov3, Mobilenet-SSD, and the improved MobileNet-SSD on the complex scene dataset. The detection of each algorithm for each category is shown in Figure 9.

**FIGURE 10:**
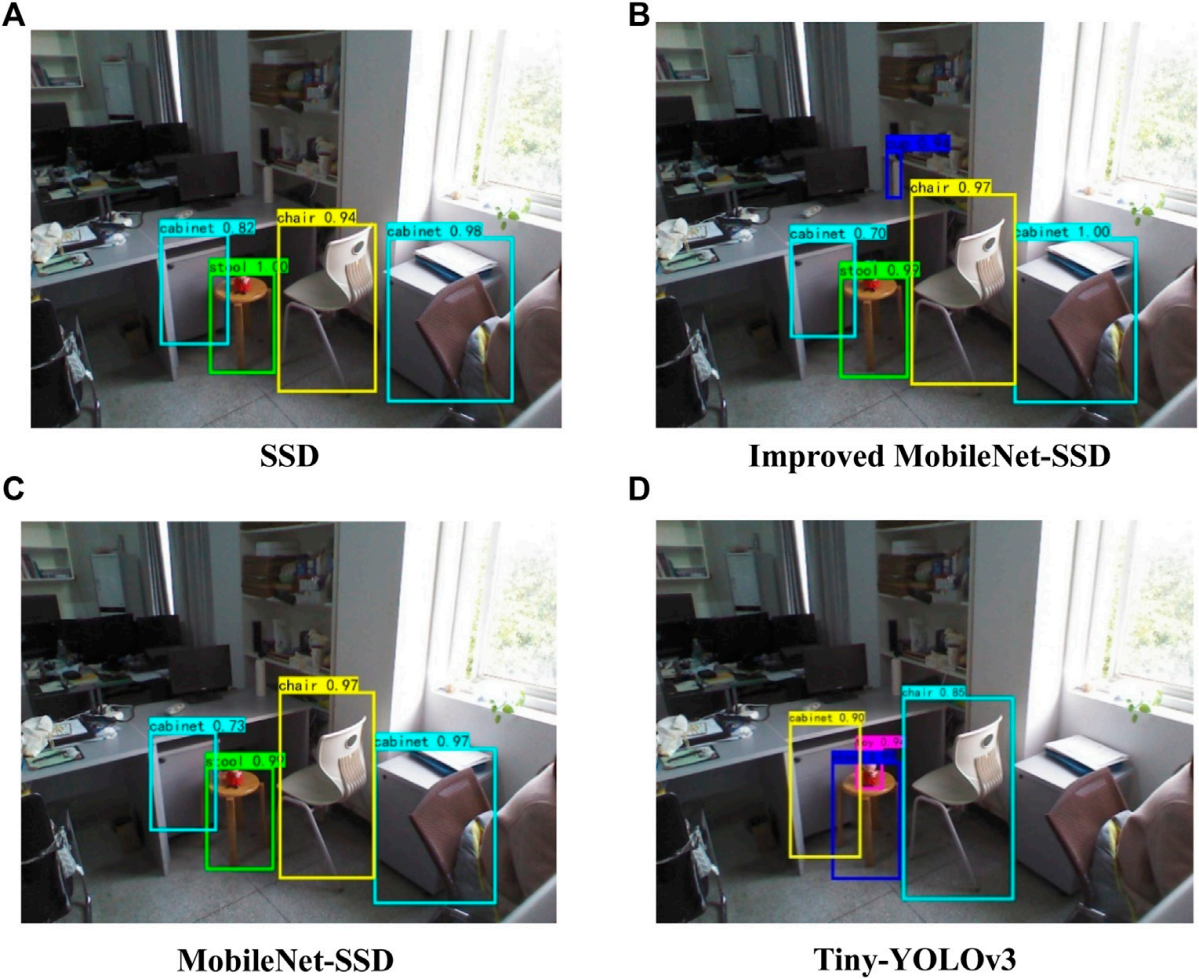
Comparison of detection effects between SSD and the lightweight target detection model. **(A)** SSD. **(B)** Improved MobileNet-SSD. **(C)** MobileNet-SSD. **(D)** Tiny-YOLOv3.

The comparison between the detection accuracy, speed, model parameters, and training time of SSD and several lightweight target detection algorithms is shown in Table 4. As can be seen from the table, compared with SSD, the detection accuracy of SSD improved by using the depthwise separable residual module was not reduced, but the number of model parameters was greatly reduced, which is conducive to model deployment, improves detection speed, and improves the real-time performance of the target detection algorithm. Compared with Mobilenet-SSD and Tiny-YOLOv3, SSD based on depthwise separable convolution had a smaller number of model parameters and a lower detection speed, but had a huge advantage in detection accuracy. When the confidence threshold is set to 0.5, the detection effect of SSD, lightweight SSD, Mobilenet-SSD, and Tiny-YOLOv3 on the same image is shown in Figure 10.

**TABLE 4:** Performance comparison between SSD and lightweight target detection algorithms.

Evaluation standard algorithm	mAP, %	FPS	MB	Training time/min
SSD	87.13	26	93.2	37
Improved MobileNet-SSD	87.33	47	27.3	12.6
Tiny-YOLOv3	66.57	52	33.2	15.3
MobileNet-SSD	67.02	62	26.8	11.4

**FIGURE 11:**
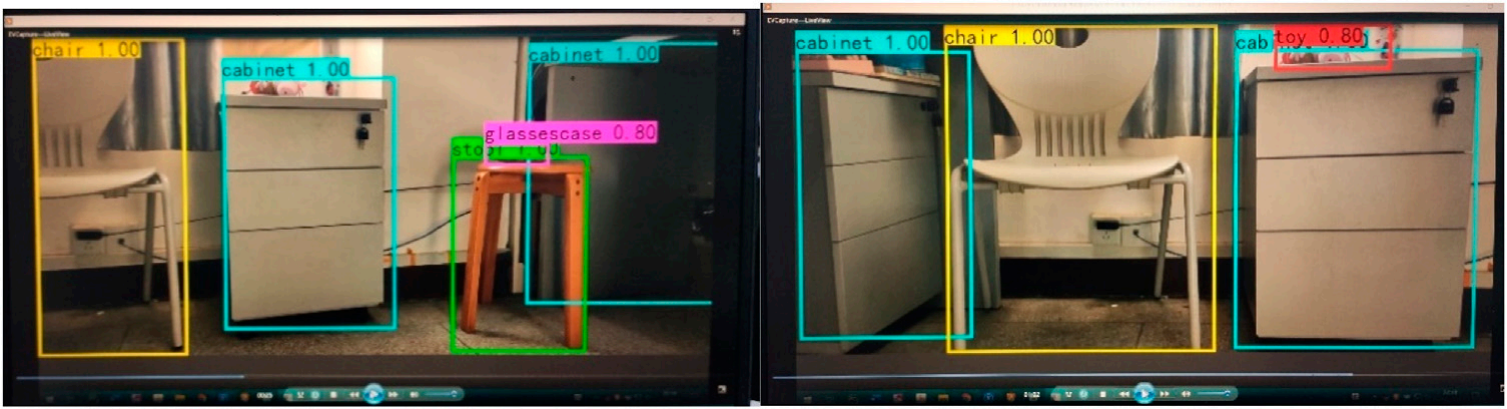
Detection effect of real-time detection model on video.

The real-time detection model was tested on video, and its detection speed met the real-time requirement. Figure 11 shows the detection effect of the real-time target detection model on video.

**FIGURE 12:**
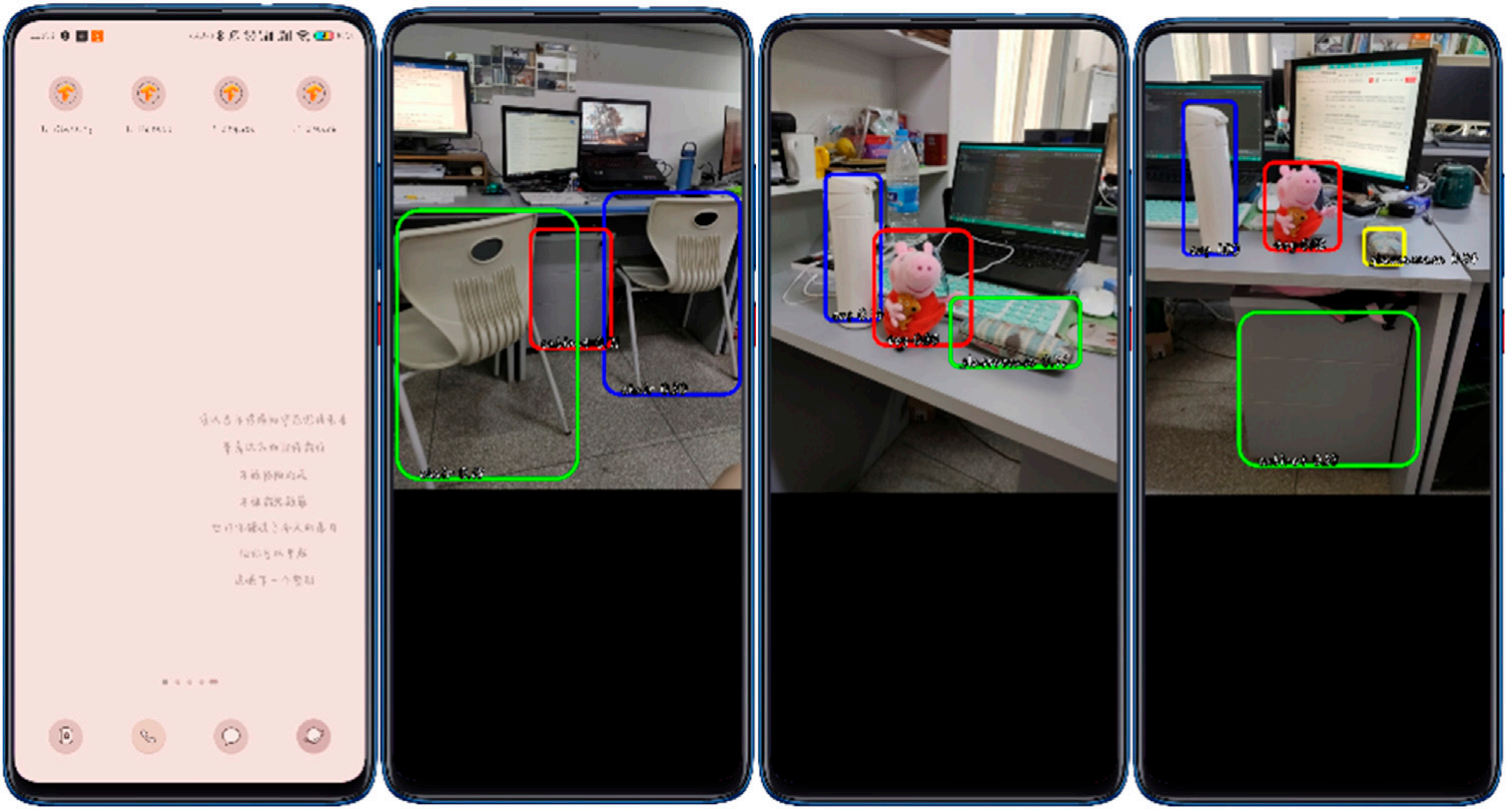
Deployment of real-time detection model on the Android platform.

It has become a trend for the model to run on the mobile terminal. In order to verify the scalability of the model, the TensorFlow model generated by Android Studio was deployed to the Android mobile terminal, the project was compiled and run, the deployment of the real-time and high-precision target detection model on the mobile end was completed, and the real-time detection on the mobile end was realized. The experimental results are shown in Figure 12.

## 5 Conclusion

In order to solve the application problem of the target detection model in embedded devices and mobile terminals, this article focuses on the research of target detection algorithm lightweight. First, the MobileNet-SSD network was introduced and analyzed, and then improved by combining the depthwise separable convolution, no-bottleneck residual module, and the convolution substitution structure to reduce parameter quantity and improve detection speed. A comparative experiment was carried out on the self-built complex scene target detection dataset; the experimental results show that the MobileNet-SSD improved relative to the SSD model precision without loss and greatly reduced the number of parameters of the model, which is advantageous to the model in the mobile terminal, deployment of embedded devices, and improvement of the detection speed of the algorithm, namely, the real-time target detection. Compared with the existing lightweight target detection network, the real-time target detection model based on the lightweight convolutional neural network proposed in this article has similar parameters, but has great advantages in detection accuracy. Finally, the model was tested on video to verify the real-time performance of the model, and the model is deployed on the Android platform to verify the scalability of the model. There are still shortcomings in this study. In future research, the neural structure search method can be used to optimize the detection speed and accuracy of the model while limiting the number of neural network parameters, so as to achieve high accuracy and real-time performance of target detection technology on embedded devices.

## Data Availability

The original contributions presented in the study are included in the article/Supplementary Material; further inquiries can be directed to the corresponding authors.
